# MRI-derived cardiac mechanical dispersion for risk stratification in patients with ischemic cardiomyopathy: a preliminary study

**DOI:** 10.1186/1532-429X-18-S1-P67

**Published:** 2016-01-27

**Authors:** Elisabeth H Paiman, Qian Tao, Alexander F Androulakis, Katja Zeppenfeld, Hildo J Lamb, Rob J van der Geest

**Affiliations:** 1 Department of Radiology, Leiden University Medical Center, Leiden, Netherlands; 2 Department of Cardiology, Leiden University Medical Center, Leiden, Netherlands

## Background

Prediction of ventricular arrhythmias (VA) after myocardial infarction remains challenging. There has been evidence that strain echocardiography can improve risk prediction. Patients with a high degree of contraction inhomogeneity are thought to be at increased risk of arrhythmic events. Mechanical dispersion may be due to inhomogeneous electrical activation and the resulting dyssynchronous contraction may further promote pathological remodelling. Aim of this study was to quantify mechanical dispersion, based on radial wall motion tracking from short-axis cine MRI, and its association with VAs and mortality.

## Methods

A total of 136 patients (age 63 ± 11 years) with ischemic cardiomyopathy underwent cardiac MRI prior to implantable cardioverter-defibrillator (ICD) placement. Outcome measure was defined as the composite of appropriate ICD therapy and all-cause mortality. On short-axis cine MRI, endo- and epicardial contours were semi-automatically tracked over the complete cardiac cycle. For each slice, radial wall motion was derived for 20 radial segments from the displacement of the endocardial contours, relative to the center of the epicardial contours. Radial wall motion curves were filtered by means of a Hanning window and clustered according to similarity of contraction patterns, to constitute a patient-specific reference curve. Delay time between the radial wall motion curve for each segment and the reference curve was calculated by cross correlation as previously described by Suever et al. [1]. Mechanical dispersion was quantified as the standard deviation of the delay time for all segments.

## Results

Median follow-up was 50 months (interquartile range 36-67 months). A total of 43 patients (32%) received appropriate ICD therapy and 21 (15%) patients died, without receiving ICD therapy before death. Univariate Cox proportional hazard analysis resulted in a hazard ratio (HR) of 1.09 per 10 ms increase of mechanical dispersion, which, however, was not statistically significant (95% CI: HR 0.99-1.20/10 ms; p = 0.07).

## Conclusions

In this preliminary study, post-infarction patients with increased mechanical dispersion as computed from cine-MRI have demonstrated a tendency, although not statistically significant, towards experiencing spontaneous VA or all-cause mortality. Further investigations are necessary to understand the potential predictive value of this mechanical parameter, with differentiation of high/low ejection fraction levels, on a larger group of patients.

**Table 1:** Baseline MRI variables

	Total population (n = 136)	No appropriate ICD therapy or all-cause mortality (n = 72)	Appropriate ICD therapy or all-cause mortality (n = 64)	P value
LVEF, %	30 ± 9	31 ± 9	28 ± 9	0.03
Mechanical dispersion, ms	61 ± 25	58 ± 23	65 ± 26	0.1

**Figure 1:**
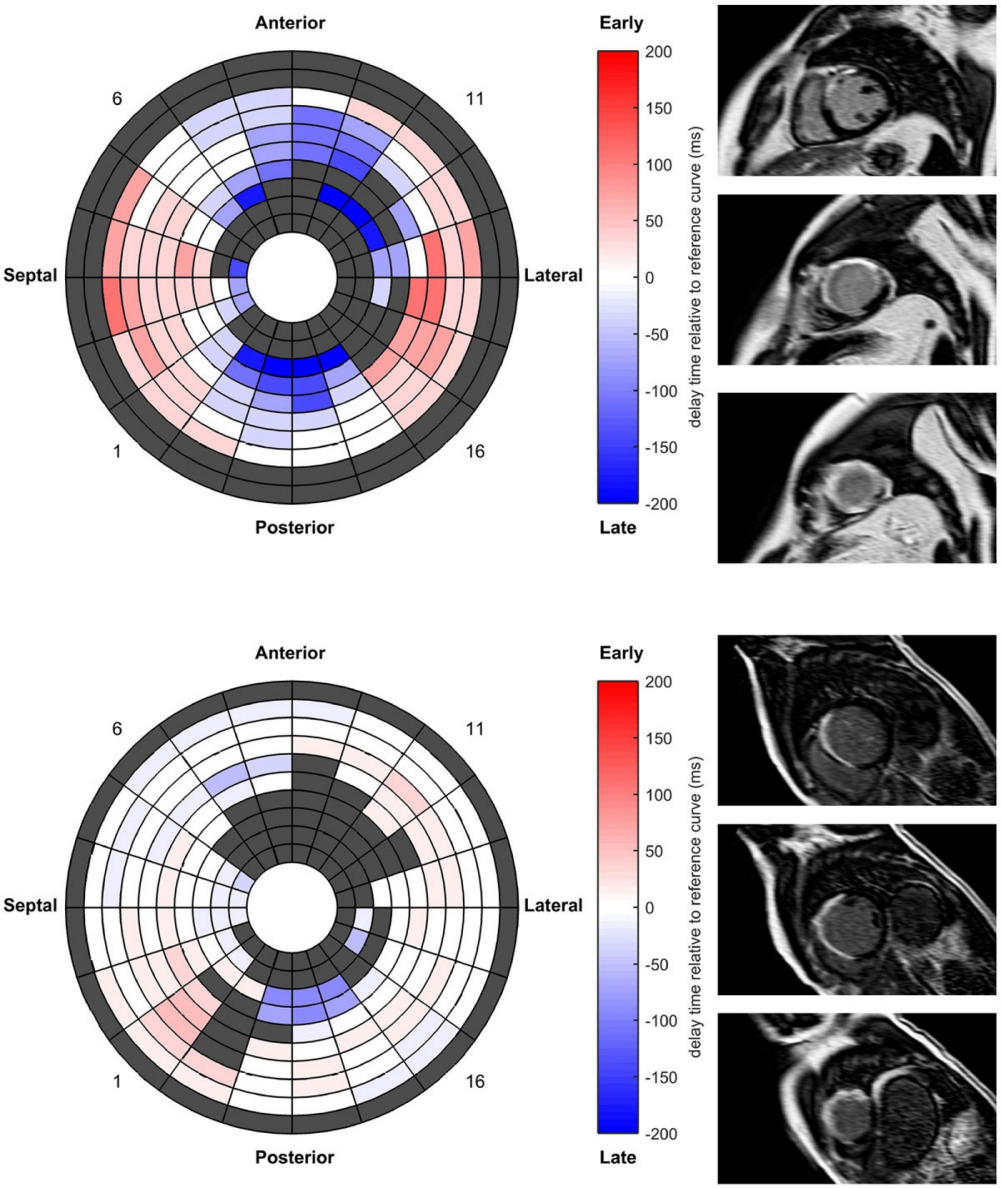
**Example of a positive case (upper panel) and a negative case (lower panel) of mechanical dispersion (83 and 26 ms, respectively) as a predictor for ventricular arrhythmia or all-cause mortality**. (left) Bullseye for regional mapping of the delay time in mechanical contraction. Non-moving segments and manually excluded slices are depicted in gray. (right) Late gadolinium enhancement MRI for viability, 3 short-axis slices. (upper panel) Patient of 41 years old, LVEF 26%, total scar mass, 57 g. Appropriate ICD therapy (shock) 5 months after MRI. (lower panel) Patient of 52 years of, LVEF 25%, total scar mass, 63 g. During the follow-up of 40 months, no arrhythmic event occurred.

